# Multimodal Imaging Characteristics of ADRP in a Family with p.Thr58Arg Substituted RHO Mutation

**DOI:** 10.1155/2020/8860863

**Published:** 2020-12-02

**Authors:** Misty Ruppert, John Pyun, K. V. Chalam, David Sierpina

**Affiliations:** Department of Ophthalmology, Loma Linda University School of Medicine, Loma Linda, CA, USA

## Abstract

**Background:**

Autosomal dominant retinitis pigmentosa (adRP) is a rare cause of progressive visual impairment in young patients and is frequently a result of RHO gene mutations. p.Thr58Arg rhodopsin mutation leads to misfolding of rhodopsin, subsequent accumulation in the endoplasmic reticulum, and leads to consecutive atrophy of photoreceptor cells through apoptosis.

**Materials and Methods:**

We describe multimodal imaging findings in a 58-year-old female with adRP due to a c.173 C > G, p.Thr58Arg rhodopsin mutation (confirmed on genotyping), including ultra-wide-field fundus autofluorescence (UWF-FAF), color scanning laser ophthalmoscopy, structural optical coherence tomography (OCT), OCT-angiography (OCT-A), electroretinography (ERG), and visual field testing (HVF). Additionally, we compare the patient's phenotypic findings to those of her offspring, who was also affected by adRP.

**Results:**

The 58-year-old female and her son with symptoms of nyctalopia and decreased vision showed macular pigmentary changes in a bull's-eye pattern along with bone spicules in periphery with retinal atrophy. Genotyping confirmed p.Thr58Arg rhodopsin mutation. Wide area of dystrophic retina was noted on UWF-FAF, along with corresponding atrophy of photoreceptor layer on OCT. OCTA revealed complete nonperfusion of the superficial capillary plexus in areas of retinal dystrophy. ERG revealed increased latency and decreased amplitudes; HVF revealed constriction of visual fields consistent with retinal findings.

**Conclusions:**

Multimodal imaging is extremely helpful in delineating the extent of retinal dystrophy and comparable to ERG for monitoring of progress in retinitis pigmentosa. Photoreceptor layer thickness (measured with OCT) strongly correlated with ERG and can be used as a secondary surrogate for monitoring the disease progress.

## 1. Introduction

Retinitis pigmentosa (RP), an inherited retinal degenerative disease, classically presents as night blindness in adolescence and progresses to complete blindness in adulthood [1]. With a prevalence of approximately 1 in 4000, RP is a common cause of blindness affecting young patients in developed countries [2, 3].

Approximately 25% of RP cases are inherited in an autosomal dominant fashion [4]. To date, 25 genes have been implicated in autosomal dominant retinitis pigmentosa (adRP) (1); nearly 30% of these cases are caused by mutations in the RHO gene (Gene ID: 6010; OMIM: 180380) located on the long arm of chromosome 3 at position 22.1 [5]. The RHO gene encodes the G-protein-coupled receptor pigment rhodopsin, which enables low-light vision by the retinal rod photoreceptor cells. RHO gene mutations are primarily missense or nonsense [6] and play a role in constitutively active rhodopsin [7]. p.Thr58Arg rhodopsin mutation (one of the rare variants) leads to misfolding of rhodopsin with subsequent accumulation in the endoplasmic reticulum and leads to consecutive atrophy of photoreceptor cells through apoptosis.

Retinitis pigmentosa is associated with typical fundus abnormalities including pigmentary changes resembling bone spicules, waxy pallor of the optic nerve, posterior sclerotic cataract formation, and attenuated retinal blood vessels [1,8]. Electroretinogram (ERG) abnormality and structural changes on optical coherence tomography (OCT) that correlate with visual acuity (VA) are characteristic [9]. Peripheral-to-central progression causing tunnel-like field of vision is common [8], though this pattern is not universal [10].

We describe multimodal imaging findings in a 58-year-old female and her son with adRP due to a c.173 C > G, p.Thr58Arg rhodopsin mutation (confirmed on genotyping), including ultra-wide-field fundus autofluorescence (UWF-FAF), color scanning laser ophthalmoscopy, structural optical coherence tomography (OCT), OCT-angiography (OCT-A), electroretinography (ERG), and visual field testing (HVF). Additionally, we compare the patient's phenotypic findings to those of her offspring.

## 2. Phenotype Description

### 2.1. Case 1

A 58-year-old female presented with complaints of progressive loss of vision and flashes of light in both eyes (OU). Her past ocular history was significant for progressive nyctalopia which was gradually worsening since her childhood. Her past medical history was significant for asthma, depression, musculoskeletal pain, well-controlled hypertension, and hyperlipidemia. Her systemic medications included hydrochlorothiazide, lisinopril, aspirin, albuterol, buspirone, duloxetine, lorazepam, gabapentin, hydrocodone-acetaminophen, tizanidine, and trazodone.

The patient's family history of adRP was further explored. Her paternal grandmother and the grandmother's two daughters were affected by RP. She has one son who is also affected with nyctalopia.

Her best-corrected visual acuity (BCVA) was 20/40 OD and 20/30 OS. Her intraocular pressure (IOP) was 20 OD and 19 OS. Anterior segment examination was unremarkable.

Fundoscopy of the right eye revealed normal optic nerve with a cup-to-disc ratio of 0.1 bilaterally. Examination of the posterior pole revealed perimacular pigmentary changes in a bull's-eye pattern. Large area of retinal atrophy in a doughnut shape was noted around vascular arcades. Attenuated vasculature was noted in the area of the dystrophic retina. Peripheral retinal examination revealed bone spicule pigmentation along the inferonasal quadrant.

Fundoscopy of OS showed similar findings. Bone spicules were noted in the inferotemporal quadrant.

### 2.2. Case 2

The patient's 38- year-old son had similar symptoms of nyctalopia since he was a child. His past medical history was unremarkable. He denied distortions, floaters, and flashing lights OU. His past ophthalmic history was unremarkable.

His BCVA was 20/40–2 OD and 20/60–2 OS. Her intraocular pressure (IOP) was 20 OD and 19 OS. Anterior segment examination was unremarkable.

Fundoscopy of OD revealed normal optic nerve with a cup-to-disc ratio of 0.1 bilaterally. The fovea was normal. Large area of retinal atrophy was noted along the inferior arcade. An attenuated vasculature was noted in the area of the dystrophic retina. Peripheral retinal examination revealed bone spicule pigmentation along the inferonasal quadrant.

Fundoscopy of OS showed similar findings. Bone spicules were noted in the inferotemporal quadrant.

## 3. Multimodal Imaging Findings

### 3.1. Color Scanning Laser Ophthalmoscopy (cSLO)

**Figure 1:**
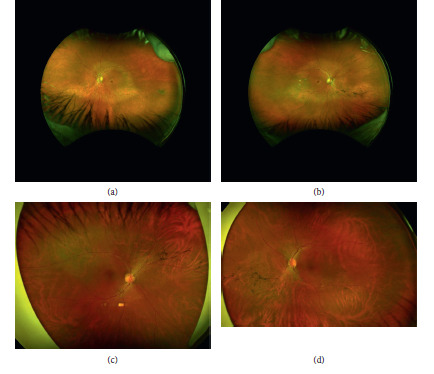
Color scanning laser ophthalmoscopy (Optos California): (a) and (b) images of the patient's right eye and left eye, respectively, showing minimal bone spicule pigmentation largely in the right eye, with circumferential midperipheral atrophy in both eyes. (c) and (d) Images of the offspring's right eye and left eye, respectively, showing similar inferonasal perivascular pigmentary clumping and bone spicule formation in both eyes.

**Figure 2:**
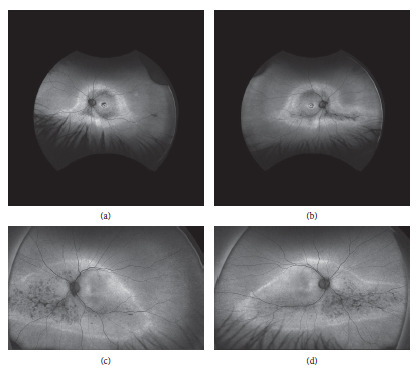
Ultra-wide-field fundus autofluorescence (UWF-FAF): (a) and (b) images of the patient's right eye and left eye, respectively, showing macular autofluorescence changes in a bull's-eye pattern OU with a midperipheral ring of hyperautofluorescence, denoting the border between the functional and dysfunctional retina. (c) and (d) Images of the offspring's right eye and left eye, respectively, lacking the bull's-eye pattern of the maculae that was present in his mother but showing a relative increase in the area of the dystrophic retina.

Color scanning laser ophthalmoscopy (Optos California) of the patient's right eye and left eye (Figures 1(a) and 1(b)), respectively, shows minimal bone spicule pigmentation largely in the right eye, with circumferential midperipheral atrophy in both eyes. In the offspring, the area of retinal dystrophy was largely confined to the inferior retina outside the vascular arcades (Figures 2(a) and 2(b)) of the offspring's right eye and left eye, respectively. In addition, there were inferonasal perivascular pigmentary clumping and bone spicule formation in both eyes.

### 3.2. Ultra-Wide-Field Fundus Autofluorescence (UWF-FAF)

Ultra-wide-field fundus autofluorescence (UWF-FAF) of the patient's right eye and left eye, respectively, show macular autofluorescence changes in a bull's-eye pattern OU with a midperipheral ring of hyperautofluorescence, denoting the border between the functional and dysfunctional retina. The atrophic area measured 160.7 sq·mm in OD and 128.4 sq·mm in OS. The hyperreflective autofluorescent area measured 160.7 sq·mm in OD and 128.4 sq·mm in OS (Figures 2(a) and 2(b)).

UWF-FAF of the offspring's right eye and left eye, respectively, lack the bull's-eye pattern of the maculae that was present in his mother but show a relative increase in the area of the dystrophic retina. The atrophic area measured 286 sq·mm in OD and 281.5 sq·mm in OS. The hyperreflective autofluorescent area measured 247.1 sq·mm in OD and 240.9 sq·mm in OS (Figures 2(c) and 2(d)).

### 3.3. OCT

**Figure 3:**
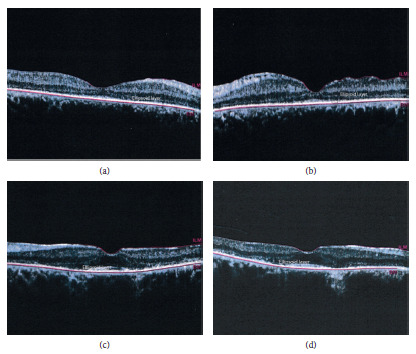
Structural OCT (Heidelberg Engineering): (a) and (b) images showing perifoveal ellipsoid zone disruption of the patient's right eye and left eye, respectively. (c) and (d) Images showing the offspring's right eye and left eye, respectively, with greater ellipsoid loss in both eyes compared to his mother.

Structural OCT (Heidelberg Engineering) revealed severe perifoveal ellipsoid zone disruption of the patient's right eye and left eye, respectively (Figures 3(a) and 3(b)). The central subfield thickness was 234 *μ* in OD and 233 *μ* in OS. The photoreceptor layer measured 75 *μ* in OD and 91 *μ* in OS.

OCT of the offspring revealed severe perifoveal ellipsoid zone disruption of the right eye and left eye, respectively (Figures 3(c) and 3(d)). The central subfield thickness was 158 *μ* in OD and 190 *μ* in OS. The photoreceptor layer measured 35 *μ* in OD and 41 *μ* in OS.

The degree of retinal atrophy was much more pronounced in the offspring compared to his mother.

### 3.4. OCT-Angiography (OCT-A)

**Figure 4:**
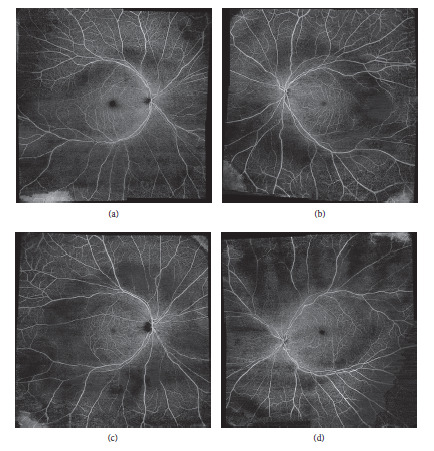
En face OCT-Angiography (Zeiss AngioPlex): (a) and (b) images of the patient's right eye and left eye, respectively, showing posterior pole montage dropout of the superficial capillary plexus (SCP) corresponding to the areas of atrophy seen on cSLO. (c) and (d) Images showing findings in the offspring's right eye and left eye, respectively.

En face OCT-Angiography (Zeiss AngioPlex), a noninvasive test, was performed to evaluate the status of retinal circulation. A large area superficial capillary nonperfusion was noted corresponding the area of retinal degeneration (Figures 4(a) and 4(b)) and measured 514 sq·mm in OD and 547 sq·mm in OS in the patient.

Similarly, OCTA examination of the offspring revealed a large area of superficial capillary nonperfusion corresponding to the area of retinal degeneration (Figures 4(c) and 4(d)) and measured 690 sq·mm in OD and 596 sq·mm in OS. Posterior pole montage was used to measure the surface area. The corresponding structural OCT showed severe atrophy of the photoreceptor layer.

### 3.5. Visual Fields

**Figure 5:**
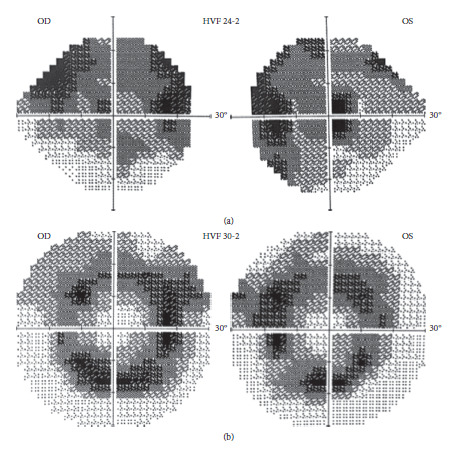
(a) A 30–2 protocol humphrey visual field of the patient that shows circular scotomas, corresponding to the midperipheral ring of hyperautofluorescence seen on UWF-FAF. (b) A 24–2 protocol humphrey visual field of the offspring showing largely superior scotomas OU, corresponding to atrophy seen on UWF-FAF and dropout of superficial capillary plexus seen on OCTA.

Humphrey Visual Field (30-2 protocol) testing revealed visual field loss (OD- 14.46 dB and OS- 13.19 dB) with generalized constriction and ring scotomas (Figure 5(a)) corresponding to the midperipheral ring of hyper autofluorescence seen on UWF-FAF.

In contrast, visual field testing revealed more severe visual field loss (OD- 15.99 dB and OS- 16.32 dB) with generalized constriction (Figure 5(b)) corresponding to the areas of retinal dystrophy seen on UWF-FAF.

### 3.6. Electroretinography

Full-field ERG of the patient and her offspring was performed. *β-*wave amplitudes were attenuated in both scotopic and photopic conditions in the mother and her offspring (Table 1).

**Figure 6:**
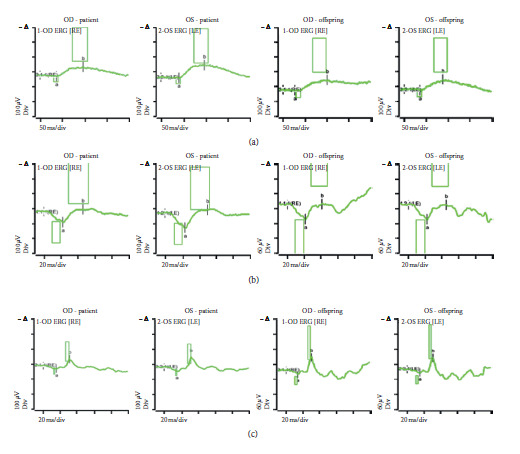
Full-field ERG of the patient and her offspring, demonstrating abnormal electroretinal function of the rod and cone systems. (a) Scotopic 0.01 averaged ERG, (b) scotopic 3.0 averaged ERG, and (c) photopic 3.0 averaged ERG.

Scotopic ERG *β*-wave amplitudes (at 0.01 ERG and 3.0 ERG scotopic modes) were 80.29 (normal 225.4 ± 101.4) and 110.47 (312.9 ± 134.1). The photopic ERG *β*-wave amplitude was 64.38 (normal 123.6 ± 50.93) in OD of the mother (Figure 6).

Scotopic ERG *β*-wave amplitudes (at 0.01 ERG and 3.0 ERG scotopic modes) were 116.8 (normal 225.4 ± 101.4) and 182.4 (312.9 ± 134.1). The photopic ERG *β*-wave amplitude was 86.7 (normal 123.6 ± 50.93) in OS of the mother.

Scotopic ERG *β*-wave amplitudes (at 0.01 ERG and 3.0 ERG scotopic modes) were 100.2 (normal 225.4 ± 101.4) and 63.1 (312.9 ± 134.1). The photopic ERG *β*-wave amplitude was 51.48 (normal 123.6 ± 50.93) in OD in the off spring.

Scotopic ERG *β*-wave amplitudes (at 0.01 ERG and 3.0 ERG scotopic modes) were 84.82 (normal 225.4 ± 101.4) and 76.8 (312.9 ± 134.1). The photopic ERG *β*-wave amplitude was 54.32 (normal 123.6 ± 50.93) in OS in the offspring.

## 4. Discussion

**Figure 7:**
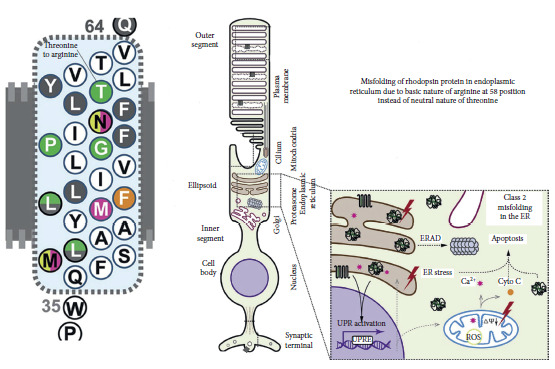
Threonine to arginine replacement is noted at position 58; cartoon documenting the pathobiology including misfolding of rhodopsin with accumulation in the endoplasmic reticulum and subsequent apoptosis of photoreceptor cells.

The c.173 > G, p.Thr58Arg mutation is a rare point mutation in codon 58 of the RHO gene, where a neutral threonine is substituted for a charged arginine in the first transmembrane domain of rhodopsin [3]. p.Thr58Arg rhodopsin mutation (due to basic nature of arginine) leads to misfolding of rhodopsin with subsequent accumulation in the endoplasmic reticulum and leads to consecutive atrophy of photoreceptor cells through apoptosis (Figure 7).

Clinical features associated with this mutation have been described by several authors [10–12]. Fishman et al. described inferior and inferonasal predilection for pigmentary changes and visual field losses primarily in the superior hemisphere in eight family members with adRP due to a p.Thr58Arg mutation [10]. Based on ERG and psychophysical threshold profiles, the p.Thr58Arg mutation was associated with a milder RP phenotypic presentation when compared with other adRP subtypes [10]. Richards et al. provided further evidence of milder adRP phenotype with this mutation, describing 16 family members who had late onset of visual symptoms and no complete blindness even with increased age [12].

The inferiorly-located bone spicule pigmentary changes seen in cSLO images of our patient and her son are consistent with previous descriptions of pigmentation in adRP patients with a p.Thr58Arg mutation [10]. Our patient displayed macular pigmentary changes in a bull's-eye pattern, which have not been previously reported in adRP due to a p.Thr58Arg mutation. Interestingly, bull's-eye pigmentary changes were present only in the patient and not in her son. In 1994, Kikawa et al. reported adRP associated with bull's-eye maculopathy, although the adRP-associated mutation was found to be an Asn244Lys mutation in the peripherin (RDS) gene [13].

Our patient's visual field losses in the form of circular scotomas differed from those seen in her son. Her findings also differ from the majority of Fishman et al.'s patients but may bear slight resemblance to one patient who displayed midperipheral partial ring scotomas with predominance in the superior field. [10]. Our patient's son had largely superior scotomas OU, a finding that has been previously noted in the majority of adRP patients with the p.Thr58Arg mutation [10]. Even with characteristic visual field losses, adRP patients with the p.Thr58Arg mutation have retained partial peripheral fields and maintained functional foveal vision in at least one eye, suggesting a favorable adRP prognosis [10].

Patients with p.Thr58Arg-associated adRP can maintain useful visual function late in life. In the family presented by Richards et al., VA remained at 20/25 or better up to 65 years of age [12]. While VA remains functionally adequate, it is expected to progressively worsen with age but has varied greatly among individuals despite wide age differences [12]. Fishman et al. reported best-corrected VAs of 20/20–2 OD and 20/30–3 OS in a 24-year-old patient, 5/600 OD and 20/50–2 OS in a 67-year-old patient, 20/20 OD and 20/20–2 OS in a 32-year-old patient, 20/30 + 2 OD and 20/30–2 OS in a 22-year-old patient, and 20/25 + 2 OD and 20/25–2 OS in a 38-year-old patient [10]. Our patient's VA of 20/40 OD and 20/30 OS was consistent with a milder presentation of adRP. Her son also maintained adequate vision in one eye of 20/40–2 OD but likely had poorer vision of 20/60–2 OS due to long-standing amblyopia.

Even though our patient and her son suffered from adRP due to the same mutation, both individuals displayed very different phenotypic outcomes. This phenotypic variability is consistent with prior reports [10,12] and leads us to conclude that one point mutation is not fully responsible for the phenotypic findings seen in Thr58Arg-associated adRP. Such variability may be due to epigenetic and environmental factors or developmental gender differences, although this remains poorly understood. It has been hypothesized that phenotypic variability may occur due to variable activity levels of photoreceptor activity [7]. It is likely that phenotypic differences are related to age.

ERG testing and *β*-wave amplitudes in full-field ERG are routinely used to monitor the progress of adRP. Multimodal imaging, a recent advance, can be useful in monitoring the progress of disease process. Photoreceptor layer thickness on OCT and superficial capillary nonperfusion directly correlated with amplitudes on scotopic ERG in our patients. OCT is commonly available in most clinics and can be used as a surrogate to ERG in monitoring the progress of the disease.

New therapies are actively being studied in an attempt to provide treatment options for patients who suffer from rhodopsin-related adRP. One clinical trial (AURORA) is currently testing intravitreal injections of QR-1123 as a treatment for adRP patients with a P23H mutation in the RHO gene [14]. QR-1123 attempts to preserve wild-type rhodopsin protein, while decreasing expression of the mutant rhodopsin protein [14]. Further reports of the phenotypic presentation and clinical course of adRP patients combined with appropriate genetic testing will allow physicians to better understand the disease progression and counsel patients and their family members accordingly.

In conclusion, we describe multimodal imaging findings in the family of adRP due to a c.173 C > G, p.Thr58Arg rhodopsin mutation (confirmed on genotyping) and compare them to electroretinography. Photoreceptor layer thickness on OCT and superficial capillary nonperfusion on OCTA can complement the ERG findings in establishing the diagnosis and can serve as secondary surrogate to ERG in monitoring the progression of retinal degeneration.

## Figures and Tables

**Table 1:** Full-field electroretinography (ERG) findings in the proband (mother) and son, all performed as per the ISCEV (International Society for Clinical Electrophysiology of Vision) standard. Flash stimuli are given in units of candela-seconds per square meter, and normative data were obtained from Diagnosys LLC.

	Mother OD	Mother OS	Son OD	Son OS	Normal range	Comments
Dark-adapted 0.01 cd∗s/m^2^*α*-wave amplitude (*μ*V)	−7.648	−6.063	−6.07	−7.459	−26.59 ± 21.72	Significant reductions in *β*-wave amplitudes suggest dysfunction at the level of the rod photoreceptor or inner retina
Dark-adapted 0.01 cd∗s/m^2^*β*-wave amplitude (*μ*V)	64.32	89	72.27	73.96	218.9 ± 117.2	

Dark-adapted 3 cd∗s/m^2^*α*-wave amplitude (*μ*V)	−72.84	−100.1	−60.21	−60.31	−142.9 ± 75.12	Attenuation of mostly *β*-wave amplitudes in both patients is indicative of nonspecific photoreceptor dysfunction
Dark-adapted 3 cd∗s/m^2^*β*-wave amplitude (*μ*V)	87.08	122.3	64.65	61.07	269.4 ± 145.4	

Dark-adapted 3 cd∗s/m^2^*α*-wave implicit time	23.3	23.3	21.63	22.46	14.87 ± 4.89	*α*-wave and *β*-wave implicit time delays are an indicator of photoreceptor dysfunction, rather than a decrease in the total number of photoreceptors. In cases of photoreceptor number reduction (e.g., retinal detachment), amplitude reduction with normal implicit times would be expected
Dark-adapted 3 cd∗s/m^2^*β*-wave implicit time	45.76	49.92	41.6	44.93	41.27s ± 11.55	

Dark-adapted 10 cd∗s/m^2^*α*-wave amplitude (*μ*V)	−87.24	−133.2	−70.42	−76.67	−191.9 ± 73.2	Reductions in *α*-wave and *β*-wave amplitudes, taken together with the *β*-wave amplitude reduction on dim flash dark-adapted full-field ERG, indicate rod photoreceptor dysfunction rather than inner retinal layer dysfunction
Dark-adapted 10 cd∗s/m^2^*β*-wave amplitude (*μ*V)	108.9	153.7	74.28	56.53	309.7 ± 136.2	

Light-adapted 3 cd∗s/m^2^*α*-wave amplitude (*μ*V)	−22.53	−27.08	−14.38	−14.17	−34.25 ± 25.13	Relative preservation of light-adapted response indicates pure rod or rod-cone dystrophy
Light-adapted 3 cd∗s/m^2^*β*-wave amplitude (*μ*V)	67.16	95.05	67.51	61.94	119.1 ± 66.48	

30 Hz flicker trough-peak amplitude (*μ*V)	62.59	77.76	53.94	45.71	108.1 ± 30.27	Attenuation of the 30 Hz flicker, taken together with the electrophysiologic findings above, allow for the diagnosis of rod-cone dystrophy in these patients

## Data Availability

Data are kept in the department of ophthalmology, Loma Linda University School of Medicine.
